# Supplementary material for: Cotranslational assembly confers specificity for in vivo target heterodimerization of paralogous H2B-like TAF12 proteins in the human fungal pathogen Candida albicans
Source: bioRxiv. 2025 Jun 26:2025.06.23.660276. Preprint. [Version 1] doi: 10.1101/2025.06.23.660276 (PMC12262449; doi:10.1101/2025.06.23.660276)
Supplement: Supplement 1 [file NIHPP2025.06.23.660276v1-supplement-1.pdf]

bioRxiv preprint doi: https://doi.org/10.1101/2025.06.23.660276; this version posted June 26, 2025. The copyright holder for this preprint (which was not certified by peer review) is the author/funder, who has granted bioRxiv a license to display the preprint in perpetuity. It is made available under aCC-BY 4.0 International license.

Supplementary information

Table S1. List of strains and plasmids

Table S2. List of oligonucleotides

Table S3: List of proteins identified by MudPIT analysis of TBP-TAP, TAF11-TAP, and TAF12L-FLAG purifications analysed by Venn diagram and the list of overlapping and non-overlapping proteins

Figure S1. *A*, Growth phenotype analysis of *TAF4* and *ADA1* depleted strains.

Figure S2. Polysome profiling of *S. cerevisiae.*

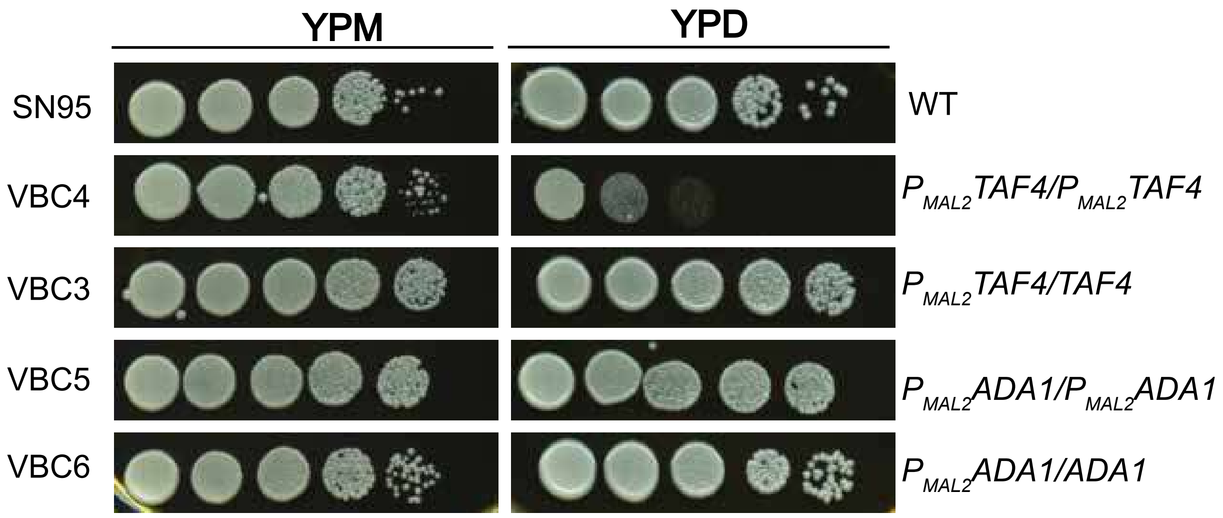

Figure S1
Bhardwaj et al.

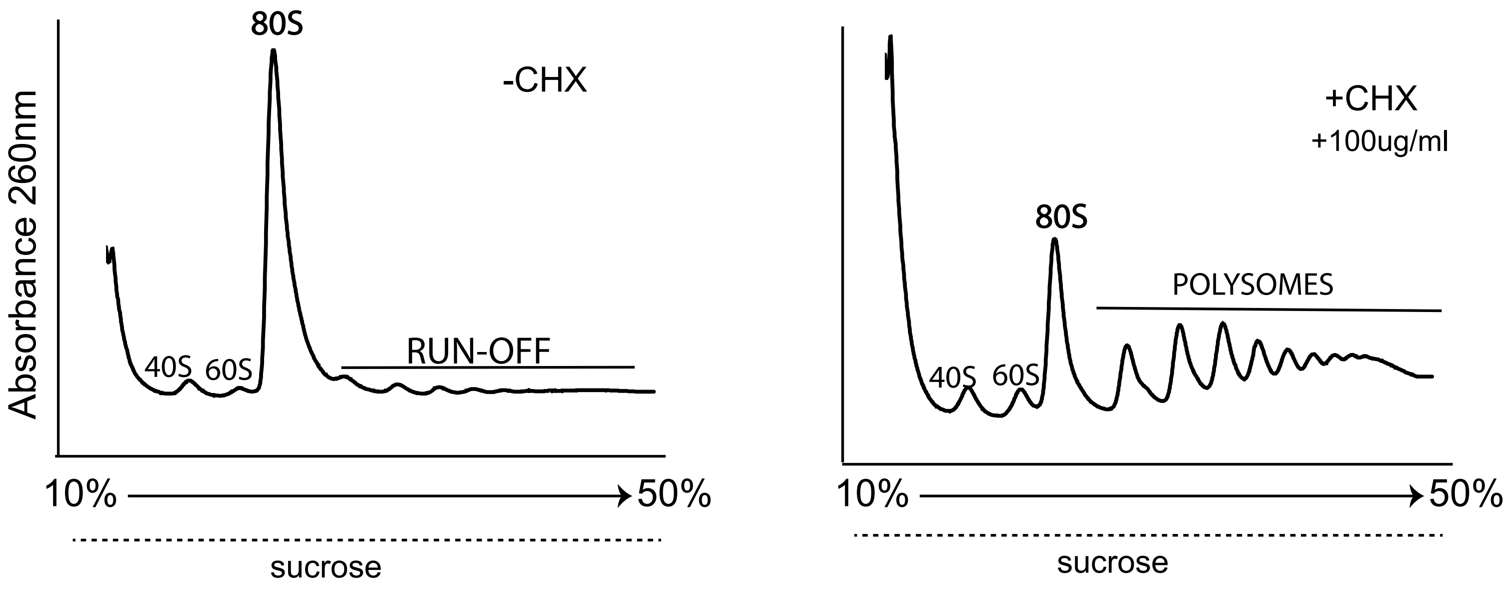

Figure S2
Bhardwaj et. al

Table S1. List of Strains

Strain	RELEVANT GENOTYPE	SOURCE
SN95	*arg4Δ/arg4Δ his1Δ/his1Δ URA3/ura3Δ::imm^{434} IRO1/iro1Δ::imm^{434}*	(Noble and Johnson, 2005)
SN87	*ura3Δ-iro1Δ::imm434/URA3-IRO1, his1Δ/his1Δ, leu2Δ/leu2Δ*	(Noble and Johnson, 2005)
ISC11	SN95 *HAH1-P_{MAL2}-TAF12L/HIS1-P_{MAL2}-TAF12L*	(Sinha et al., 2017)
ISC12	SN95 *HAH1-P_{MAL2}-TAF12/HIS1-P_{MAL2}-TAF12*	(Sinha et al., 2017)
SKC3	SN87 *TAF12L::His6-Gly2-FLAG3-FRT/TAF12 L::His6-Gly2-FLAG3-SAT1-FLP*	(Sinha et al., 2017)
SKC6	SN87 *TAF12:: His6-Gly2-FLAG3-FRT/TAF12 ::His6-Gly2-FLAG3-SAT1-FLP*	(Sinha et al., 2017)
SKC8	SN87ADA1::His6-Gly2-FLAG3-FRT/ADA1	(Sinha et al., 2017)
SKC12	SN87 TAF4::His6-Gly2-FLAG3-FRT/TAF4::His6-Gly2-FLAG3-SAT1-FLP	(Sinha et al., 2017)
VBC3	*SN95 P_{MAL2}TAF4/TAF4*	This work
VBC4	*SN95 P_{MAL2}TAF4/P_{MAL2}TAF4*	This work
VBC5	*SN95 P_{MAL2}ADA1/PADA1*	This work
VBC6	*SN95 P_{MAL2}ADA1/P_{MAL2}ADA1*	This work

Table S2. List of Oligonucleotides

OLIGO NUMBER	LENGTH	SEQUENCE (5' to 3')	NOTES
ONC114	18	5'-GGTGCCACTGATCCATTG-3'	Position 61 to 78 within *CaARG4* ORF
ONC115	18	5'-GCCAACATATCCATAGTTAAAGC-3'	Position 1108 to 1130 within CaARG4 ORF
ONC116	21	5'-CCGTATTCCATGATTGCTATG-3'	Diagnostic primer within CaMAL2 promoter; position -230 to -210 upstream of target genes ATG.
ONC104	18	5'-TAAAAATATCGCACTCAC-3'	3'-primer for *ADH1;* +1374 to +1356 wrt ATG
ONC114	18	5'-GGTGCCACTGATCCATTG-3'	Position 61 to 78 within Ca*ARG4ORF* (Ca*ARG4*-F61)
ONC115	23	5'-GCCAACATATCCATAGTTAAAGC-3'	Position 1108 to 1130(c) within *CaARG4ORF* (Ca*ARG4*-R1130)
ONC123	18	5'-GCCCTTCTGCCTGGAGTA-3'	Diagnostic PCR primer within the non-repeat sequence of *HIS1* ofpHAH
ONC 400	25	5'-CAGAGAAAGCGGAGGAAAATAGTAA-3'	Forward RT primer for TAF12
ONC 401	25	5'-ATAATGGTTTGGGTTTCGACTTAGA-3'	Reverse RT primer for TAF12
ONC 431	23	5'-AGTCGAAAGAAAATTGGCTGCTA-3'	Forward RT primer for RPS8A
ONC 432	24	5'-CAGAACCGAATTGAGAGTCAACAG-3'	Reverse RT primer for RPS8A

ONC 439	21	5'-GGGCTGCAGCATTACTTTTAG-3'	Forward RT primer for ADA1
ONC 440	21	5'-GCCGTCGAATCTGTTTTGTTT-3'	Reverse RT primer for ADA1
ONC 520	21	5'-CAACTGGACGCTCAGGAACTC-3'	Forward RT primer for TAF12L
ONC 521	20	5'-GACGTGTAATGCCAGCCAAA-3'	Reverse RT primer for TAF12L
ONC 616	21	5'-GCCTGAACCGACGCAAACTTG -3'	Upcheck Forward primer ADA1 for screening
ONC 617	25	5'-GATTGCTCAAGAATTGATCCAGACC -3'	DownCheck Reverse primer for ADA1 for screening
ONC 667	21	5'-CTCATCAAAGGACTCGGGAAA-3'	Forward primer specific for CaADA1 ORF
ONC 1060	76	5'TTAAGGAAAACTCAATTATTAACAATCTTGAAATCACCGAATTATA TCACATAAATCCCTTCGTACGCTGCAGGTC3'	Forward long primer for TAF4 promoter replacement through HAH
ONC 1061	88	5’GGAGTCCTCACTGTTTTCTAATTGTCTCTTCAAATTAGAGGATTC TTGAGGTGTACTTGTCATTGTAGTTGATTATTAGTTAAACCAC3’	Reverse long primer for TAF4 promoter replacement through HAH
ONC 1062	78	5’TTCCCCCCAATAATACCTTTTATTTTGGCAATTTACATTTGAGTTA CTATTTCTTTGATTCTTCGTACGCTGCAGGTC3’	Forward long primer for ADA1 promoter replacement through HAH
ONC 1063	85	5'TCCATTTTTCAAAGGATTGATAGTAGTTGTAGAAGATCCATCAGC GATTTGAGATGTCATTGTAGTTGATTATTAGTTAAACCAC 3'	Reverse long primer for ADA1 promoter replacement through HAH
ONC 1294	20	5’-TTTGGAAGCAACACTGGACA-3’	Downcheck Reverse primer for TAF4
ONC1300	22	5’-GACTATGAATCCCGGGAGAAAG-3'	Forward RT primer for TAF4
ONC 1301	21	5’-TTTCCCGAGTCCTTTGATGAG -3'	Reverse RT primer for TAF4

bioRxiv preprint doi: https://doi.org/10.1101/2025.06.23.660276; this version posted June 26, 2025. The copyright holder for this preprint (which was not certified by peer review) is the author/funder, who has granted bioRxiv a license to display the preprint in perpetuity. It is made available under aCC-BY 4.0 International license.

Supplementary figure legends

Figure S1. ***A*****, Growth phenotype analysis of *TAF4* and *ADA1* depleted strains.** *A,* Growth phenotype of *TAF4* and *ADA1* depleted strains. Strains were grown in YPM till saturation and serially diluted, spotted on YPM and YPD plates, and incubated at 30°C. Plates were imaged at 36 h**.**

Figure S2: Polysome profiling of *S. cerevisiae*: Cell extracts from untreated(left) or CHX-treated (100µg/ml, right) *S. cerevisiae* BY4741 cultures fractionated through a 10% to 50% sucrose gradient.

bioRxiv preprint doi: https://doi.org/10.1101/2025.06.23.660276; this version posted June 26, 2025. The copyright holder for this preprint (which was not certified by peer review) is the author/funder, who has granted bioRxiv a license to display the preprint in perpetuity. It is made available under aCC-BY 4.0 International license.

Table S3

Input for Venn Diagram

TBP-TAP			TAF12L-FLAG			TAF11-TAP		
NAME	TBP-TAP dNSAF AVG	Seq Coverage	NAME	TAF12L-FLAG dNSAF AVG	Seq Coverage	NAME	TAF11-TAP dNSAF AVG	Seq Coverage
TBP1	0.10922	82.77	C1_10620W	0.17428	74.74	TAF4	0.084788	26.17
HSP70	0.10276	67.07	GAR1	0.06072	48.92	CR_03460W	0.081502	59.62
C7_00340C	0.09018	58.81	NOP10	0.05025	67.8	TAF60	0.080882	53.18
RIM1	0.04008	49.65	RPL4B	0.03364	46.28	CR_04450C	0.078424	42.48
C7_01400C	0.0376	66.19	RPL43A	0.03011	39.13	TAF145	0.063528	46.07
C4_04600C	0.03529	59.49	RPL25	0.0239	46.48	TAF10	0.062019	54.1
TRI1	0.03478	42.06	RPL10A	0.02346	43.32	C2_02500W	0.057825	35.31
C5_03830C	0.02275	35.5	RPL2	0.02273	35.43	C3_03930W	0.039747	31.03
TAF60	0.0213	63.97	RPP2A	0.02005	54.63	TAF12	0.035394	21.87
HHF22	0.01831	61.9	RPP0	0.01938	44.87	C5_03830C	0.03287	26.02
SSA2	0.01807	72.56	BBC1	0.01705	58.18	RPS7A	0.015967	30.74
RPL4B	0.01599	30.3	ACS1	0.01658	59.7	RPP2A	0.011951	31.72
CR_04450C	0.01585	52.26	RPL8B	0.01496	59.92	HSP70	0.011874	31.48
CR_04310C	0.01574	65.64	RPL3	0.01459	43.44	TBP1	0.008814	35.37
TAF10	0.01319	75	RPL39	0.01438	32.32	RPL12	0.00795	26.98
CR_03460W	0.01274	61.26	CR_04450C	0.01383	61.65	YDJ1	0.006466	35.71
TAF145	0.01252	51.78	ADA2	0.0117	53.93	SIS1	0.006218	20.61
TAF4	0.01212	39.39	RPP1A	0.01141	20.75	TAF14	0.004786	36.64
RPP2A	0.01198	47.22	RPL10	0.01135	46.36	TEF1	0.004786	36.64
C2_02500W	0.00982	37.69	RPL12	0.0104	67.88	PDA1	0.004736	24.78
RPL8B	0.0079	42.75	NGG1	0.01003	65.07	RPL30	0.004551	31.18
TAF12	0.00711	44.27	CR_10450C	0.00981	39.94	RPL18	0.004293	19.21
RPL12	0.00628	63.03	CR_04870C	0.00962	59.28	C3_04380C	0.004051	27.18
RPL43A	0.00603	19.57	SPT7	0.00901	63.35	C2_05710C	0.004033	28.3
RPL18	0.00577	22.04	SSB1	0.00802	54.81	RPL10	0.003677	20.43
TAF14	0.00548	36.12	GCN5	0.0076	37.19	RPP2B	0.003393	19.05
RPL14	0.00536	37.4	NHP2	0.00751	49.69	RNR1	0.003196	17.76
RPL6	0.00504	51.7	RPS12	0.00709	55.24	TDH3	0.003109	18.18
C2_07190C	0.00489	41.38	RPL15A	0.00641	32.84	SMT3	0.003081	15.32
C3_03930W	0.00485	32.07	RPS6A	0.00595	30.08	C2_03160C	0.002703	22.95
TEF1	0.00428	31.22	SSC1	0.0056	54.94	KAR2	0.002552	25.37
TOA2	0.00427	40.77	RPL18	0.00545	30.65	TUB1	0.002515	10.78
RPL10	0.00403	31.82	RPL23A	0.00527	35.04	C2_07190C	0.00249	24.27
YDJ1	0.00402	44.02	SPT20	0.00481	42.74	RPL6	0.002489	16.59
C1_00710C	0.00395	42.09	C2_05830C	0.00479	61.3	RPP1A	0.002481	16.52
HTA2	0.00395	29.77	C7_00450C	0.00471	53.21	SDH12	0.002457	13.79
C1_04180W	0.0037	11.54	RPS8A	0.00445	50.49	RIM1	0.002429	8.52
RPL10A	0.00358	33.64	TAF60	0.0044	57.61	RPC10	0.00242	14.15
RPL30	0.00349	57.55	RPS3	0.00427	33.07	TAF19	0.002401	11.39
RPL3	0.00333	36.76	RPL14	0.00417	34.35	C1_00160C	0.002391	12.59
SIS1	0.00313	45.48	C5_02900W	0.00413	48.96	SSB1	0.002342	27.4
C2_05710C	0.00311	17.76	RPL24A	0.00403	24.52	C4_04160W	0.002332	13.94
SMT3	0.0029	32.35	RPL32	0.00402	23.66	RPL2	0.002318	13.86
RPL2	0.00262	19.29	C3_03100C	0.00382	55.51	C1_00710C	0.002137	15.5
RPL39	0.00262	23.23	SBP1	0.0038	45.74	VMA2	0.002092	20.72

bioRxiv preprint doi: https://doi.org/10.1101/2025.06.23.660276; this version posted June 26, 2025. The copyright holder for this preprint (which was not certified by peer review) is the author/funder, who has granted bioRxiv a license to display the preprint in perpetuity. It is made available under aCC-BY 4.0 International license.

RPL15A	0.00254	21.08	C2_05710C	0.00365	26.17	SIK1	0.002024	14.79
KAR2	0.00251	35.08	TAF12L	0.00362	44.27	SBP1	0.00202	10.63
SSB1	0.00244	41.6	TAF10	0.0032	59.43	MRP7	0.001904	16.48
RPP2B	0.00233	40.93	C5_03830C	0.0031	35.5	TSM1	0.001837	10.16
C2_10680W	0.00211	20.72	RPS14B	0.0031	43.94	GPM1	0.001823	20.74
RPP1A	0.00209	12.08	TEF1	0.00307	38.65	CMD1	0.001819	19.86
HTA1	0.00196	40.2	HHF22	0.00297	43.81	ERG13	0.001758	20.82
VMA2	0.00195	14.15	C1_00180W	0.00293	30.5	RPS13	0.00174	14.39
BRF1	0.00194	39.19	RPS15	0.00289	42.25	C1_00900W	0.001724	19.35
UBI3	0.00192	28.03	RPL6	0.00288	41.48	RPL8B	0.001721	30.2
RPP0	0.00178	27.93	ERG13	0.00277	37.92	RPS25B	0.001706	14.41
TIF	0.00177	21.16	ACT1	0.00275	55.03	RPP1B	0.001699	19.21
RPL24A	0.00167	8.29	RPL28	0.00275	33.24	RPL10A	0.001668	7.8
TUB1	0.00165	33.01	TRA1	0.00272	44.57	SKP1	0.001632	20.99
RPL27A	0.00163	30.48	C1_00710C	0.00265	42.09	KGD2	0.001628	12.38
RPL35	0.00154	12.9	C2_03560C	0.00263	58.73	TIF	0.001583	14.81
EFT2	0.00154	31.7	RPL19A	0.00257	21.05	NOP5	0.001576	11.98
RPS18	0.00153	19.85	RPL9B	0.00255	43.98	C1_12280C	0.001564	10.37
RPL28	0.00149	16.67	C3_04380C	0.00248	36.51	RPS26A	0.001551	10.88
TAF19	0.00148	26.48	RPL27A	0.00244	43.38	C4_04390W	0.001507	15.11
RPS1	0.00144	19.31	RPS18	0.00242	36.55	GAR1	0.001491	12.4
RPL19A	0.00136	26.85	RPS23A	0.00242	31.72	C7_00790W	0.001474	11.64
TSM1	0.0013	30.3	C6_02310W	0.0023	50.64	C1_03370W	0.001437	12.61
C1_00180W	0.00129	21.05	C3_07050W	0.0023	67.23	PDC11	0.001379	14.52
C4_05820W	0.00126	26.17	RPL30	0.00221	57.55	PDB1	0.001379	16.94
C1_05720W	0.00123	23.68	RVB1	0.00217	41.7	C1_04180W	0.001379	14.65
PIL1	0.0012	11.4	RPL13	0.00212	28.22	RPL14	0.001371	16.15
RPS21	0.00119	33.12	C3_05790C	0.00211	13.33	RPS14B	0.001359	13.2
C3_04380C	0.00117	11.5	CR_04240C	0.00208	41.75	BMH1	0.001357	13.76
C1_02330C	0.00117	27.27	UBI3	0.00202	32.64	RPL27A	0.001353	8.18
RPL21A	0.00116	27.39	CR_04110W	0.00202	49.4	C1_04180W	0.001315	11.54
SKP1	0.00113	23.38	RPS21B	0.00202	18.39	C2_00360C	0.001305	9.16
RPL23A	0.00108	19.68	RPS19A	0.00202	33.79	RPS12	0.001295	13.64
RPS8A	0.00108	19.05	NOP5	0.00197	31.2	RPS19A	0.001295	20.08
PDB1	0.00107	15.75	RPL35	0.00195	25	ENO1	0.001257	6.62
C1_00590W	0.00104	24.38	C2_07190C	0.00191	41.38	C7_02660C	0.001227	6.22
RPL25	0.00104	20.73	MRT4	0.00187	38.26	RVB1	0.001223	15.59
PDA1	0.00101	25.55	RPL21A	0.00183	25.62	RPP0	0.001196	21.68
SIK1	0.001	23.79	C1_11080W	0.00173	45.43	C4_05630W	0.001179	11.72
RPL5	0.00099	17.41	RPS25B	0.00167	21.9	C7_03000C	0.001166	16.14
MRT4	0.00097	17.84	RPS10	0.00165	41.53	C1_05720W	0.001135	11.5
C5_01540W	0.00095	18.31	RPS16A	0.00165	44.37	HEM1	0.00112	15.28
RPS20	0.00093	25.19	RPS1	0.0016	34.38	MGE1	0.001096	12.5
LAT1	0.00093	24.22	RPS24	0.00159	25.93	HSP60	0.001075	10.69
SBP1	0.00092	21.48	RPP2B	0.00158	20.72	ATP2	0.001069	17.5
HSP104	0.00091	37.76	SIK1	0.00155	43.6	RPS42	0.001064	10.37
ERG13	0.0009	30.12	TEF2	0.00153	38.65	TIF34	0.001061	7.62
C4_04820C	0.00086	21.3	HSP70	0.00151	35.37	HSP90	0.00106	7.02
RPL20B	0.00086	24.52	RPS28B	0.00146	37.31	C6_04290W	0.001057	10.25
C2_03950W	0.00084	10.92	RPP1B	0.00145	14.81	NSP1	0.001018	9.52

bioRxiv preprint doi: https://doi.org/10.1101/2025.06.23.660276; this version posted June 26, 2025. The copyright holder for this preprint (which was not certified by peer review) is the author/funder, who has granted bioRxiv a license to display the preprint in perpetuity. It is made available under aCC-BY 4.0 International license.

PPH21	0.00082	14.47	TUB1	0.00144	37.95	HSP21	0.000979	7.63
RPS24	0.00082	14.18	VMA2	0.00141	47.07	RPL19A	0.000977	11.14
CR_05150W	0.00081	19.69	ASC1	0.00135	38.49	RPL9B	0.000967	8.91
RPS9B	0.00078	20.62	TDH3	0.00128	47.46	UBI3	0.000962	8.04
MRPL19	0.00078	13.18	CR_04390C	0.00128	20.88	CHC1	0.000953	6.69
RPS12	0.00078	15.7	RPL17B	0.00127	21.08	TIF4631	0.000905	5.29
C2_07680W	0.00077	16.98	MRP7	0.00125	36.5	SSA2	0.0009	8.95
C1_00160C	0.00074	10.9	PR26	0.00123	28.95	C1_00180W	0.000895	6.28
RPL13	0.00073	10.28	RPS5	0.00121	13.78	RPS8A	0.000886	8.29
C1_00900W	0.00072	18.52	C5_01700W	0.00121	30	CR_08290W	0.000876	12.23
CR_10350C_	0.00072	14.29	C1_04180W	0.0012	19.23	HHF22	0.000868	7.84
PTC2	0.0007	7.41	ADH1	0.00117	42.29	ASC1	0.000862	24.34
C2_04570W	0.00069	12.59	C1_00900W	0.00114	23.41	C7_00340C	0.000862	24.34
RPP1B	0.00069	23.78	RPL11	0.00112	24.71	URA2	0.000855	6
C2_03560C	0.00069	20.21	C4_04390W	0.0011	47.58	C2_04120C	0.00083	8.74
C1_14500C	0.00067	29.82	C4_03040W	0.00108	44.44	MRT4	0.00082	4.8
C4_03090W	0.00067	15.93	C1_03620C	0.00105	23.08	CR_06800C	0.000814	12.38
C4_04160W	0.00066	13.75	HTA2	0.00104	35.88	ADH1	0.000809	9.15
C2_01740C	0.00065	10.89	TIF	0.00103	31.23	RPS6A	0.000792	11.64
RNR1	0.00065	23.41	C2_07680W	0.00102	20.21	LAT1	0.000772	9.48
RVB1	0.00065	24.27	RPS20	0.00098	10.92	TPI1	0.000752	6.59
MRPL3	0.00064	21.1	C1_05720W	0.00097	18.67	RVB2	0.000743	6.52
GFA1	0.00062	17.1	BMH1	0.00096	29.92	RPS21	0.000737	14.66
C5_01050C	0.00062	14.81	RPS9B	0.00093	17.46	C4_03410W	0.000733	9.71
PET9	0.00061	18.78	EFT2	0.00088	22.57	RPS1	0.000725	5.08
CR_00460C	0.00061	5.91	C2_05410W	0.00087	12.5	C4_04820C	0.000717	8.6
RPL17B	0.0006	11.27	YML6	0.00084	18.35	RPL3	0.000689	8.87
PRT1	0.0006	17.36	C5_00820W	0.00083	45.23	SDH2	0.000687	6.02
GPM1	0.0006	10.62	MRPL37	0.00081	13.33	MET3	0.000687	10.04
RFA1	0.00059	8.67	GFA1	0.0008	28.75	HTA1	0.000686	16.15
C1_03370W	0.00059	10.26	IMG2	0.00079	34.15	VMA8	0.000668	6.64
KGD2	0.00059	15.35	C4_02260C	0.00079	26.68	C5_00030W	0.000663	5.81
CR_03120W	0.00057	15.29	RPS21	0.00078	39.76	C2_10680W	0.000659	6.94
C2_04120C	0.00057	12.78	RVB2	0.00078	25.9	DBP5	0.00065	4.18
RPL32	0.00057	11.96	C1_02330C	0.00077	18.9	C5_00150C	0.000649	6.64
HET1	0.00056	8.2	HSP60	0.00076	21.55	C5_04720C	0.000648	22.73
RPS14B	0.00056	15.68	C5_01540W	0.00076	18.06	YML6	0.00064	7.49
BMH1	0.00056	10.69	C7_03000C	0.00073	23.75	MNT1	0.000636	7.06
GLC7	0.00056	13.31	GPM1	0.00071	20.16	SUB2	0.000636	11.66
TDH3	0.00055	20.83	RPT6	0.00068	20.7	C4_04330C	0.000633	6.85
C5_00030W	0.00055	9.86	RPL20B	0.00068	25	PET9	0.00063	6.81
RPC40	0.00055	10.88	RPT5	0.00068	25.58	C1_01860W	0.000629	5.51
DBP5	0.00055	15.53	CR_10820W	0.00067	28.45	RPL24A	0.000615	3.96
URA2	0.00053	8.79	AHP1	0.00067	50	SHM2	0.000595	5.34
PDX1	0.00053	7.63	RPL5	0.00066	17.11	C7_00070C	0.000592	7.85
MCI4	0.00053	17.26	C6_02470W	0.00065	29.92	MDJ1	0.000568	3.99
HSP90	0.00052	13.64	ATP1	0.00064	16.67	C5_02210W	0.000568	5.98
RPS15	0.00052	34.09	UBP8	0.00064	21.37	SGT2	0.000561	6.56
C4_03410W	0.00051	14.85	RPS7A	0.00063	17.2	HSP104	0.000552	7.1
CIC1	0.00051	17.01	C1_03370W	0.00062	25.12	PPH21	0.000546	6.81

bioRxiv preprint doi: https://doi.org/10.1101/2025.06.23.660276; this version posted June 26, 2025. The copyright holder for this preprint (which was not certified by peer review) is the author/funder, who has granted bioRxiv a license to display the preprint in perpetuity. It is made available under aCC-BY 4.0 International license.

ACS2	0.00049	13.01	PDC11	0.00062	23.99	C4_06680C	0.000539	5.68
DCW1	0.00049	8.33	CR_07220C	0.00062	19.52	RPL4B	0.000526	5.53
RPS13	0.00049	16.11	C2_04120C	0.0006	15.16	CR_00460C	0.000523	6.12
C3_01720C	0.00049	10.38	TSA1B	0.0006	20.41	C3_00450C	0.000497	5.23
C1_09620C	0.00048	5.28	RVS161	0.00059	18.18	PRT1	0.000476	5.67
SHM2	0.00047	8.63	C6_03430C	0.00059	22.13	MP65	0.000475	5
CR_10830C_	0.00047	8.46	C4_03410W	0.00058	16.15	PFK2	0.000472	3.53
ASC1	0.00047	15.28	HSP90	0.00058	20.23	C2_03560C	0.000471	5.23
C5_04990W	0.00045	13.38	C1_00110W	0.00058	24.44	RPS9B	0.000467	8.2
MRPL40	0.00044	13.4	SSA2	0.00058	42.95	LAB5	0.000465	4.62
C5_00560W	0.00044	12.33	RPS17B	0.00057	30.66	C2_03360W	0.000463	3.79
C1_01680C	0.00044	15.24	CR_10350C	0.00057	24.27	C1_06590C	0.000452	4.23
C1_01370C	0.00043	7.52	CR_07080W	0.00056	16.33	SLK19	0.000452	5.82
RPL11	0.00043	7.95	C5_01050C	0.00054	13.33	C1_11200W	0.000452	3.28
RPS42	0.00042	12.69	C1_12610W	0.00053	16.82	MRPL3	0.000452	5.03
ENO1	0.00042	9.66	RPS42	0.00052	29.39	RPL15A	0.000443	4.92
SDH12	0.0004	9.74	C3_06970W	0.00052	13.37	CAM1	0.00044	2.06
C4_06730C	0.0004	10	C5_00030W	0.00051	13.01	CDC48	0.000432	7.32
C6_03380W	0.0004	7.59	CDC19	0.0005	30.16	LYS22	0.000429	5.37
C7_01210C	0.0004	14.2	RNR1	0.0005	18.62	NOG1	0.000429	2.34
RPS7A	0.0004	10.43	RPT4	0.0005	17.99	C2_04570W	0.000423	4.95
HSP21	0.00039	10.65	C5_04910W	0.00049	18.14	C6_02690C	0.000419	5.88
C4_05900C	0.00038	9.47	RPS26A	0.00049	12.61	C1_12610W	0.000415	5.1
RPL7	0.00038	8.82	ENO1	0.00049	16.36	NUP49	0.000414	4.12
C5_02380W	0.00037	18.39	C5_02660C	0.00047	20.6	ACC1	0.00041	3.36
MET10	0.00037	8.05	RSM22	0.00046	12.02	C6_00290W	0.000401	5.63
RVB2	0.00037	11.45	C2_03950W	0.00044	20	C2_02170W	0.0004	5.62
PWP1	0.00036	3.82	RPT1	0.00044	15.32	C1_05270C	0.000398	5.59
CR_07320C	0.00036	16.14	CR_00460C	0.00043	8.2	C1_10620W	0.000389	6.82
MAK16	0.00035	6.71	RPN13	0.00042	19.49	CR_07080W	0.000381	2.23
NOP1	0.00035	6.99	C1_05270C	0.00041	25.94	LPD1	0.000376	5.81
C7_00790W	0.00035	13.59	SAM2	0.00041	18.44	CDC53	0.000375	2.92
SRP40	0.00035	6.12	YDJ1	0.0004	22.14	CDC19	0.000373	3.71
GSP1	0.00035	15.59	ADE4	0.0004	28.84	C3_05880C	0.000358	4.6
CHC1	0.00033	5.29	RPS13	0.00039	19.21	MTS1	0.000357	2.71
KGD1	0.00033	12.76	SMT3	0.00038	21.57	RTG3	0.000349	9.39
C2_05410W	0.00033	5.76	SRB1	0.00038	9.67	C3_03410C	0.000348	2.65
CR_10740W	0.00033	10.05	C4_05630W	0.00037	16.04	CCR4	0.000341	4.39
C2_07370W	0.00032	8.23	PDB1	0.00036	18.47	HEM14	0.000339	2.38
C1_11200W	0.00032	7.43	C2_05160C	0.00035	14.69	SSZ1	0.000335	3.73
C1_12280C	0.00032	13.05	C5_04630W	0.00035	16.96	C3_00850C	0.000333	3.31
RPS6A	0.00031	6.8	MRPL40	0.00035	21.3	PDI1	0.000329	4.42
C5_04910W	0.00031	8.25	C2_01740C	0.00035	10.62	EFT2	0.000327	4.49
RPS26A	0.00031	8.23	ACS2	0.00034	11.98	MPP10	0.000326	3.56
DPM1	0.00031	4.7	C1_12280C	0.00034	11.64	NOT4	0.000322	3.01
MGE1	0.00031	9.11	RPT2	0.00034	15.52	CR_00490W	0.000317	3.33
PRX1	0.0003	6.54	ACH1	0.00034	8.4	PES1	0.000314	1.96
CR_07080W	0.0003	10.9	TRP99	0.00032	7.07	C4_03290W	0.000306	2.33
LPD1	0.0003	14.16	PFK26	0.00032	20.92	C5_02380W	0.000305	5.34
ARO8	0.0003	12.5	KAR2	0.00031	12.95	AAF1	0.000302	3

bioRxiv preprint doi: https://doi.org/10.1101/2025.06.23.660276; this version posted June 26, 2025. The copyright holder for this preprint (which was not certified by peer review) is the author/funder, who has granted bioRxiv a license to display the preprint in perpetuity. It is made available under aCC-BY 4.0 International license.

IMG2	0.0003	4.44	CR_07510W	0.00031	8.8	MIR1	0.000297	3.3
NSA2	0.00028	6.98	PRE8	0.00031	6.37	PIL1	0.000292	6.14
SDH2	0.00028	9.88	RHR2	0.00031	10.24	C2_05300C	0.000291	3.41
NOP15	0.00028	5.17	C1_00160C	0.00029	14	LSP1	0.000286	3.85
RFA2	0.00027	7.2	NOG2	0.00029	12.2	C3_02350W	0.000286	1.78
MGM101	0.00027	6.75	C3_01720C	0.00029	10.73	DED1	0.000279	2.78
HEM1	0.00026	7.56	C3_06760W	0.00028	4.36	ILV2	0.000278	5.52
HSP60	0.00026	4.6	PGK1	0.00028	11.51	DRG1	0.000278	5.54
ZUO1	0.00026	7.02	ALI1	0.00028	10.04	IDH2	0.000272	5.41
NSP1	0.00026	4.94	C1_11880W	0.00028	7.14	C4_02260C	0.00027	5.36
RPT5	0.00026	15.92	C2_04570W	0.00027	9.37	C5_03550W	0.000256	2.85
SUB2	0.00026	8.76	GCD2	0.00027	8.6	FLO8	0.000254	1.93
RPD31	0.00026	8.35	UBC4	0.00027	18.37	C4_00420C	0.00025	2.78
C6_00550W	0.00025	10.57	PRT1	0.00026	9.88	C2_04370W	0.000232	3.8
C4_06680C	0.00025	5.36	C4_06210C	0.00026	12.55	PGK1	0.000232	4.88
C6_02350C	0.00024	4.18	TKL1	0.00026	6.06	PDX1	0.00023	5.39
SEC24	0.00024	7.42	RPN1	0.00026	11.28	PIN4	0.000216	1.39
C1_03280W	0.00024	4.87	C2_02270C	0.00025	9.03	C1_08180C	0.000216	2.02
CR_04170W	0.00024	11.4	RNH1	0.00025	15.45	UTP9	0.000211	3.82
YTM1	0.00024	4.36	LAT1	0.00025	9.64	ROM2	0.000209	3.41
SNF12	0.00024	3.37	NIP1	0.00025	9.27	SER33	0.000205	3.6
C1_00930C	0.00024	10.42	ARO8	0.00024	13.85	SNF12	0.000205	4.32
C4_05630W	0.00023	3.28	TPI1	0.00024	8.87	ARO8	0.000199	1.97
CDC28	0.00023	7.21	GSY1	0.00024	19.52	KGD1	0.000195	1.21
LSP1	0.00023	9.05	ALD5	0.00024	14.43	CAP1	0.000192	3.6
C7_03000C	0.00023	7.16	C1_05650W	0.00023	8.73	C3_00130C	0.000186	1.16
C6_02690C	0.00023	7.8	C7_03830C	0.00023	8.33	C1_12030W	0.000185	6.48
ACC1	0.00023	7.97	C1_00590W	0.00023	14.08	YWP1	0.000182	2.99
C4_00420C	0.00023	4.08	C1_00040W	0.00023	12.94	ADE4	0.000174	3.67
MDJ1	0.00023	3.1	C4_04820C	0.00023	8.14	SRV2	0.000172	2.01
C5_03980W	0.00022	6.79	C7_03660C	0.00023	9.84	MLS1	0.000171	3.01
GCD2	0.00022	5.48	C5_03410C	0.00023	5.2	CR_02610C	0.000163	2.1
ILV2	0.00022	3.72	YST1	0.00022	17.24	UBR1	0.000161	3.2
NOP5	0.00022	7.08	VMA6	0.00022	14.37	KRE30	0.00016	3.19
AHA1	0.00021	7.46	TAF14	0.00022	13.31	C1_01590C	0.000158	2.59
C2_01390W	0.00021	6.66	C1_01580W	0.00022	6.82	HIS7	0.000157	3.12
ADH1	0.00021	5.66	LYS22	0.00022	12.23	PGA63	0.000155	2.9
MET3	0.00021	4.73	C5_00150C	0.00022	13.63	SSD1	0.000148	5.21
YWP1	0.00021	3.47	IDH1	0.00021	11.54	C6_00170C	0.000144	2.24
FBA1	0.00021	6.25	CCT7	0.00021	12.39	TKL1	0.00014	4.93
TIF4631	0.00021	7.92	PGI1	0.00021	5.82	ACS2	0.000139	1.05
C1_05630C	0.0002	4.71	NOG1	0.00021	6.72	PAN1	0.000136	3.19
TCP1	0.0002	7.27	RPN12	0.00021	11.91	TRP5	0.000135	2.69
GAR1	0.0002	5.94	HSP21	0.00021	5.29	C1_00930C	0.000134	1.57
RPG1A	0.0002	4.97	SAR1	0.00021	10.53	UTP13	0.00013	3.8
C7_02960C_	0.0002	6.8	CEF3	0.0002	7.14	NAN1	0.000126	5.18
PDC11	0.0002	9.22	C1_01600W	0.0002	10.24	C1_04290C	0.000126	2.95
RPL9B	0.00019	10.85	RPL7	0.0002	10.85	NIP1	0.000122	2.71
VMA5	0.00019	8.09	MRPL3	0.00019	11.39	RPG1A	0.000122	1.36
MRP7	0.00019	12.31	THS1	0.00019	11.05	SEC24	0.000121	1.56

bioRxiv preprint doi: https://doi.org/10.1101/2025.06.23.660276; this version posted June 26, 2025. The copyright holder for this preprint (which was not certified by peer review) is the author/funder, who has granted bioRxiv a license to display the preprint in perpetuity. It is made available under aCC-BY 4.0 International license.

GPD1	0.00018	3.26
C5_00260W	0.00018	3.14
NSA1	0.00018	6.83
AAF1	0.00018	3.19
PGK1	0.00018	4.46
CR_10490W	0.00018	3.51
LYS22	0.00018	5.42
C5_01700W	0.00018	6.49
C1_03790C	0.00017	6.49
ABP1	0.00017	11.83
SSC1	0.00017	4.09
C2_09660W	0.00017	4
C6_00170C	0.00017	7.41
C1_12610W	0.00017	6.28
PRO2	0.00017	3.87
DED1	0.00017	3.08
NUP49	0.00017	3.97
TKL1	0.00016	5.69
RPC53	0.00016	2.96
C2_02170W	0.00016	6.37
RPD3	0.00015	3.6
PHO23	0.00015	5.04
C1_10620W	0.00015	6.7
C6_02370C	0.00015	10.48
C1_09040C	0.00015	5.16
DIP2	0.00015	4.78
VID27	0.00015	4.78
CDC19	0.00015	7.34
RHR2	0.00015	4.71
C3_05880C	0.00015	5
FUN12	0.00015	3.59
C3_07460W	0.00014	5.95
RTG3	0.00014	3.79
C3_00130C	0.00014	2.66
RVS161	0.00014	3.32
ADE4	0.00014	3.71
C5_04720C	0.00014	3.75
C3_00850C	0.00014	3.34
ATP1	0.00014	2.7
CCT7	0.00014	2.07
RPN13	0.00013	7.19
ALI1	0.00013	3.67
CTA7	0.00013	2.15
RAD52	0.00013	2.38
RET2	0.00013	14.57
C1_13320C	0.00013	3.73
CR_02610C	0.00013	2.85
CSI2	0.00013	4.04
NIP1	0.00013	3.27
PES1	0.00013	4.76

PIL1	0.00019	6.82
CR_07320C	0.00019	9.39
RNR21	0.00019	10.17
PDX1	0.00019	11.51
HIS7	0.00019	6.06
GLN3	0.00019	20.67
NOP1	0.00019	5.38
C2_09660W	0.00018	5.5
C1_14500C	0.00018	6.36
UTP18	0.00018	8.7
C7_03840W	0.00018	6.04
UTP9	0.00018	9.89
C1_10470W	0.00017	12.5
CLC1	0.00017	7.56
FAS1	0.00017	6.53
SER33	0.00017	11.27
RPD31	0.00017	9.72
C6_02880W	0.00017	4.74
URA2	0.00017	8.71
FBA1	0.00016	11.98
CIC1	0.00016	4.66
C6_03380W	0.00016	4.35
C3_00450C	0.00016	8.6
ZCF29	0.00016	8.42
PMM1	0.00016	5.56
UTP5	0.00015	8.81
SDH12	0.00015	7.33
NSA2	0.00015	5.36
C2_07220W	0.00015	16.67
NOP15	0.00015	4.87
VMA8	0.00015	7.49
C3_04810C	0.00015	7.87
CR_02420W	0.00015	5.95
RPN2	0.00014	7.14
CCJ1	0.00014	7.3
MRPL10	0.00014	3.89
MYO2	0.00014	6.73
SUB2	0.00014	11.78
DPS1-1	0.00014	8.3
RAS1	0.00014	4.14
PES1	0.00013	5.79
CR_00490W	0.00013	4.78
UTP4	0.00013	10.09
PET9	0.00013	7.97
C3_06700C	0.00013	6.85
RPG1A	0.00013	9.66
YTM1	0.00013	7.2
SEC27	0.00013	8.84
C5_00080C	0.00013	10.61
PFK2	0.00012	5.07

SEC27	0.000107	1.5
C1_07340W	0.000107	4.59
C1_00960C	0.000102	2.98
GLT1	0.000099	1.27
SLA2	0.000098	1.6
SRO77	0.000092	1.83
C1_05630C	0.000092	1.72
C6_03460W	0.000091	3.62
RPA135	0.000084	1.28
RGA2	0.000082	1.15
C1_06550W	0.00008	0.8
NGG1	0.00008	2.16
SPT6	0.000079	3.07
ECM17	0.000077	1.8
MLP1	0.000074	1.89
FAS1	0.000073	2.21
C7_00570W	0.000073	1.46
C1_11860W	0.00007	1.56
C1_03290W	0.000068	3.03
C7_04300W	0.000061	3.03
C2_06170C	0.00006	1
C4_01060W	0.000047	0.77
C5_00190C	0.000042	0.76

bioRxiv preprint doi: https://doi.org/10.1101/2025.06.23.660276; this version posted June 26, 2025. The copyright holder for this preprint (which was not certified by peer review) is the author/funder, who has granted bioRxiv a license to display the preprint in perpetuity. It is made available under aCC-BY 4.0 International license.

MIR1	0.00012	7.95	CR_10830C	0.00012	11.39
SDS24	0.00012	7.58	C2_07290W	0.00012	7.23
RPN1	0.00011	4.78	UTP13	0.00012	6.38
C5_02660C	0.00011	4.29	CR_03120W	0.00012	4.97
C1_10470W	0.00011	2.38	HXK2	0.00012	5.99
FAS1	0.00011	3.28	C5_04990W	0.00012	11.04
MSI3	0.00011	4.33	C3_07420W	0.00012	5.79
CR_04240C	0.00011	5.02	CDC48	0.00012	5.45
TRP5	0.00011	2.04	C3_02350W	0.00012	8.55
C3_01850W	0.00011	3.55	RPA135	0.00012	4.03
CDC10	0.0001	4.89	MSS116	0.00012	4.64
GZF3	0.0001	2.44	RPC40	0.00012	5.06
SRO77	0.0001	9.38	ATP2	0.00012	6.75
ILS1	0.0001	9.22	MRPS9	0.00012	6.25
IDH2	0.0001	7.88	CSH1	0.00012	5.64
UTP4	0.0001	1.6	ILV2	0.00011	5.56
ACT1	9.8E-05	5.45	GRP2	0.00011	15.84
ARP7	9.8E-05	5.54	ARC35	0.00011	5.49
HOG1	9.8E-05	2.87	NMD3	0.00011	5.19
C5_03000C	9.7E-05	6.45	C5_00790C	0.00011	4.01
SAM2	9.6E-05	6.27	C3_01850W	0.00011	3.42
CR_05550C	9.6E-05	5.36	CHA1	0.00011	10.2
C2_03360W	9.5E-05	3.24	CDC10	0.00011	5.04
C1_06590C	9.3E-05	11.3	KRR1	0.00011	3.63
SLK19	9.3E-05	2.28	CHC1	0.00011	5.18
FLO8	9.3E-05	1.54	TCP1	0.00011	5.42
SEC18	9.3E-05	4.56	DRG1	0.00011	4.08
RDH54	9.3E-05	2.71	LYS21	0.00011	10.05
CDC48	0.00009	3.64	AGO1	0.00011	5.78
C2_04370W	0.00009	2.39	C6_04100W	0.00011	5.15
CAM1	0.00009	2.88	GCN1	0.00011	4.8
PR26	0.00009	2.76	C5_04640C	0.0001	8.52
CR_08290W	8.9E-05	4.61	ERG6	0.0001	5.32
SMC1	8.9E-05	4.04	ARP7	0.0001	5.29
RPT4	8.6E-05	4.5	HOG1	0.0001	4.24
DNM1	8.5E-05	2.12	HET1	9.9E-05	10.15
C1_02240W	8.5E-05	4.26	RNR22	9.9E-05	4.82
PMA1	8.3E-05	1.84	PDA1	9.7E-05	4.74
CR_03220C	8.3E-05	5.97	C1_00930C	9.7E-05	4.82
MCM2	8.2E-05	2.51	C5_00260W	9.7E-05	3.71
CDC54	8.1E-05	1.8	NSA1	9.6E-05	2.96
SER33	0.00008	4.55	LMO1	9.5E-05	5.54
SEC27	7.9E-05	2.42	GLT1	9.2E-05	4.84
FAS2	7.9E-05	1.64	TIF4631	0.00009	2.12
SEC21	7.9E-05	4.65	HSP104	8.7E-05	3.45
PFK2	7.8E-05	1.01	SWD1	8.7E-05	4.92
SEC26	7.8E-05	1.94	SES1	8.5E-05	6.33
ECM17	7.7E-05	4.63	SPT6	8.4E-05	3.5
C2_04620W	7.6E-05	4.62	SNF12	8.3E-05	7.25
PNG2	7.5E-05	2.67	C1_12240C	8.3E-05	3.4

bioRxiv preprint doi: https://doi.org/10.1101/2025.06.23.660276; this version posted June 26, 2025. The copyright holder for this preprint (which was not certified by peer review) is the author/funder, who has granted bioRxiv a license to display the preprint in perpetuity. It is made available under aCC-BY 4.0 International license.

PFK1	7.5E-05	4.8
ATP2	7.3E-05	0.97
MTS1	7.2E-05	1.71
C1_00060W	7.2E-05	2.19
C2_06980W	7.2E-05	2.23
CR_10470C_	0.00007	3.36
SLA2	0.00007	1.22
C4_06210C	0.00007	4.06
CEF3	0.00007	6.48
MLS1	6.7E-05	1.6
UTP18	6.7E-05	1.59
RPA190	6.7E-05	2.14
PGI1	6.7E-05	3.7
PDI1	6.6E-05	1.16
C2_02540W	6.4E-05	3.27
ELF1	6.2E-05	3.81
KRE30	6.1E-05	1.32
HIS7	5.9E-05	2.23
C6_04290W	5.9E-05	2.98
C3_05360C	5.9E-05	8.31
TOM1	5.6E-05	2.34
C3_02350W	5.5E-05	3.31
UTP8	5.3E-05	7.23
GLT1	5.2E-05	3.42
C4_07060W	5.1E-05	2.1
CDC53	4.9E-05	3.2
CR_03200C	4.7E-05	2.92
NAN1	4.4E-05	2.72
ERB1	4.4E-05	3.27
UBR1	4.1E-05	2.18
RPN2	3.9E-05	2.33
DCK1	3.9E-05	2.61
C1_07340W	3.6E-05	2.09
PIM1	3.4E-05	2.63
C6_03460W	3.2E-05	2.87
RGA2	3.1E-05	1.77
C1_11860W	0.00003	1.11
C1_01590C	0.00003	7.04
PGA63	2.9E-05	1.49
SSD1	2.9E-05	1.8
C1_08180C	2.8E-05	2.44
SPT6	2.6E-05	1.27
MLP1	0.00002	19.05
GCN1	1.5E-05	0.9
MDN1	1.5E-05	2.39
TRA1	0.00001	1.93

MIS11	8.2E-05	3.91
LPD1	7.9E-05	10.39
C6_03440W	7.6E-05	4.43
ARO9	7.5E-05	6.88
AIP2	7.4E-05	3.8
HOM3	7.2E-05	3.13
PBS2	7.2E-05	2.94
NAN1	0.00007	3.93
GAD1	6.9E-05	4.23
HAS1	6.9E-05	3.54
CSI2	6.7E-05	3.42
GGA2	6.7E-05	2.91
PWP1	6.4E-05	3.59
C6_02350C	6.4E-05	3.1
APE2	6.3E-05	3.79
PGA63	6.2E-05	4.35
C2_05300C	6.2E-05	5.41
SEC26	6.1E-05	3.15
ACC1	0.00006	3.43
C1_00640C	0.00006	2.77
C2_06200C	0.00006	1.83
GLC7	5.9E-05	4.24
ROB1	5.9E-05	2.12
DED1	5.8E-05	5.06
RGD1	5.8E-05	2.95
VPS1	5.6E-05	2.45
PPH21	5.4E-05	5.28
PIM1	5.4E-05	3.25
C1_08110W	5.4E-05	4.95
C1_05630C	5.3E-05	3.25
MSN4	5.2E-05	2.25
C7_03160W	5.2E-05	2.15
VMA5	0.00005	3.87
CR_03200C	0.00005	1.8
RRP6	0.00005	5.73
NUP82	4.9E-05	5.26
C2_04370W	4.8E-05	3.41
SRP40	4.6E-05	6.78
C1_03790C	4.6E-05	4.93
SEC12	4.6E-05	2.26
DNM1	4.5E-05	1.73
C1_02240W	4.5E-05	2.17
PRO2	4.4E-05	3.59
PMA1	4.4E-05	5.25
C1_14080W	4.3E-05	3.14
SHM2	4.1E-05	2.98
RPD3	4.1E-05	3.75
C6_02370C	0.00004	2.9
C1_09040C	0.00004	3.29
PNG2	0.00004	1.83

bioRxiv preprint doi: https://doi.org/10.1101/2025.06.23.660276; this version posted June 26, 2025. The copyright holder for this preprint (which was not certified by peer review) is the author/funder, who has granted bioRxiv a license to display the preprint in perpetuity. It is made available under aCC-BY 4.0 International license.

CAP1	3.9E-05	2.81
C1_00060W	3.8E-05	2.73
SRV2	3.6E-05	2.39
CRM1	3.6E-05	2.97
PEX1	3.6E-05	1.74
RPA190	3.5E-05	2.58
CCT6	3.5E-05	3.76
C5_02380W	3.3E-05	2.68
PYC2	3.3E-05	2.63
KRE30	3.2E-05	1.97
C1_01590C	3.2E-05	3.16
C7_01030C	3.2E-05	1.73
MSI3	2.8E-05	3
UTP8	2.8E-05	2.73
C2_00360C	2.8E-05	1.86
CDC53	2.6E-05	1.86
C4_00420C	2.4E-05	2.47
TOM1	2.4E-05	1.58
ERB1	2.3E-05	2.24
SNF2	2.3E-05	1.18
NAB3	2.3E-05	2.39
FAS2	2.1E-05	0.8
SPO72	2.1E-05	0.7
PFK1	0.00002	1.82
CDC39	1.9E-05	0.69
MET10	1.8E-05	1.46
SSN6	1.8E-05	1.48
MDN1	1.6E-05	1.33
C2_02540W	1.1E-05	0.93

bioRxiv preprint doi: https://doi.org/10.1101/2025.06.23.660276; this version posted June 26, 2025. The copyright holder for this preprint (which was not certified by peer review) is the author/funder, who has granted bioRxiv a license to display the preprint in perpetuity. It is made available under aCC-BY 4.0 International license.

Common elements in TAF12L-FLAG TBP-TAP TAF11-TAP :	Common elements in TBP-TAP TAF11-TAP :	Common elements in TAF12L-FLAG TBP-TAP :	Common elements in TAF12L-FLAG TAF11-TAP	TAF12L only	TBP only	TAF11 only
C1_10620W	TBP1	RPL43A	NGG1	NOP10	C7_01400C	C2_03160C
GAR1	C7_00340C	RPL25	RPS19A	BBC1	C4_04600C	RPC10
RPL4B	RIM1	RPL39	RPS25B	ACS1	TRI1	CMD1
RPL10A	CR_03460W	SSC1	C4_04390W	ADA2	CR_04310C	C7_02660C
RPL2	TAF145	RPL23A	YML6	CR_10450C	TOA2	TIF34
RPP2A	TAF4	RPL32	C4_02260C	CR_04870C	BRF1	CR_06800C
RPP0	C2_02500W	RPS15	C1_05270C	SPT7	C4_05820W	MNT1
RPL8B	TAF12	ACT1	TPI1	GCN5	CR_05150W	C4_04330C
RPL3	C3_03930W	RPL28	C5_00150C	NHP2	MRPL19	C1_01860W
CR_04450C	SIS1	TRA1	NOG1	SPT20	PTC2	C7_00070C
RPP1A	C2_10680W	RPS18	UTP9	C2_05830C	C4_03090W	C5_02210W
RPL10	HTA1	RPL13	C3_00450C	C7_00450C	RFA1	SGT2
RPL12	TAF19	CR_04240C	VMA8	RPS3	MCI4	MP65
SSB1	TSM1	RPL35	CR_00490W	C5_02900W	DCW1	LAB5
RPS12	SKP1	RPL21A	UTP13	C3_03100C	C1_09620C	C6_00290W
RPL15A	C4_04160W	RPS24	RPA135	TAF12L	C5_00560W	C3_03410C
RPS6A	KGD2	RPL17B	DRG1	RPS23A	C1_01680C	CCR4
RPL18	DBP5	PR26	C2_05300C	C6_02310W	C1_01370C	HEM14
RPS8A	C7_00790W	C5_01700W	CAP1	C3_07050W	C4_06730C	SSZ1
TAF60	KGD1	RPL11	SRV2	C3_05790C	C7_01210C	MPP10
RPL14	C1_11200W	HTA2	C2_00360C	CR_04110W	C4_05900C	NOT4
RPL24A	MGE1	C2_07680W		RPS21B	MAK16	C4_03290W
SBP1	SDH2	RPS20		C1_11080W	GSP1	C5_03550W
C2_05710C	HEM1	C2_05410W		RPS10	CR_10740W_A	PIN4
TAF10	NSP1	GFA1		RPS16A	C2_07370W	ROM2
C5_03830C	C4_06680C	IMG2		TEF2	DPM1	C1_12030W
RPS14B	SEC24	C1_02330C		RPS28B	PRX1	PAN1
TEF1	LSP1	C5_01540W		CR_04390C	RFA2	C1_04290C
HHF22	C6_02690C	RPL20B		RPS5	MGM101	C1_00960C
C1_00180W	MDJ1	RPT5		C4_03040W	ZUO1	C1_06550W
RPL6	MET3	RPL5		C1_03620C	C6_00550W	C7_00570W
ERG13	YWP1	ATP1		C5_00820W	C1_03280W	C1_03290W
C1_00710C	AAF1	RVS161		MRPL37	CR_04170W	C7_04300W
C2_03560C	C6_00170C	CR_10350C_B		RPT6	CDC28	C2_06170C
RPL19A	NUP49	C5_01050C		CR_10820W_A	C5_03980W	C4_01060W
RPL9B	C2_02170W	RPT4		AHP1	AHA1	C5_00190C
C3_04380C	C3_05880C	C5_04910W		C6_02470W	C2_01390W	
RPL27A	RTG3	C5_02660C		UBP8	C7_02960C_A	
RPL30	C3_00130C	C2_03950W		CR_07220C	GPD1	
RVB1	C5_04720C	RPN13		TSA1B	CR_10490W_A	
UBI3	C3_00850C	SAM2		C6_03430C	ABP1	
NOP5	CR_02610C	MRPL40		C1_00110W	RPC53	
C2_07190C	MIR1	C2_01740C		RPS17B	PHO23	
MRT4	TRP5	RHR2		C3_06970W	DIP2	
RPS1	SRO77	C3_01720C		RSM22	VID27	

bioRxiv preprint doi: https://doi.org/10.1101/2025.06.23.660276; this version posted June 26, 2025. The copyright holder for this preprint (which was not certified by peer review) is the author/funder, who has granted bioRxiv a license to display the preprint in perpetuity. It is made available under aCC-BY 4.0 International license.

RPP2B	IDH2	ALI1		RPT1	FUN12	
SIK1	C2_03360W	GCD2		SRB1	C3_07460W	
HSP70	C1_06590C	C4_06210C		C2_05160C	CTA7	
RPP1B	SLK19	RPN1		C5_04630W	RAD52	
TUB1	FLO8	C1_00590W		RPT2	RET2	
VMA2	CAM1	CCT7		ACH1	C1_13320C	
ASC1	CR_08290W	PGI1		TRP99	SDS24	
TDH3	ECM17	CEF3		PFK26	GZF3	
MRP7	MTS1	RPL7		CR_07510W	ILS1	
C1_04180W	SLA2	CR_07320C		PRE8	C5_03000C	
ADH1	MLS1	NOP1		NOG2	CR_05550C	
C1_00900W	PDI1	C2_09660W		C3_06760W	SEC18	
TIF	C6_04290W	C1_14500C		C1_11880W	RDH54	
C1_05720W	UBR1	UTP18		UBC4	SMC1	
BMH1	C1_07340W	C1_10470W		C2_02270C	CR_03220C	
RPS9B	C6_03460W	RPD31		RNH1	MCM2	
EFT2	RGA2	FBA1		GSY1	CDC54	
RPS21	C1_11860W	CIC1		ALD5	SEC21	
RVB2	SSD1	C6_03380W		C1_05650W	C2_04620W	
HSP60	C1_08180C	NSA2		C7_03830C	C2_06980W	
C7_03000C	MLP1	NOP15		C1_00040W	CR_10470C_B	
GPM1		RPN2		C7_03660C	ELF1	
RPS7A		UTP4		C5_03410C	C3_05360C	
C1_03370W		YTM1		YST1	C4_07060W	
PDC11		CR_10830C_A		VMA6	DCK1	
C2_04120C		CR_03120W		C1_01580W		
C4_03410W		C5_04990W		IDH1		
HSP90		RPC40		RPN12		
SSA2		C3_01850W		SAR1		
CR_07080W		CDC10		C1_01600W		
C1_12610W		TCP1		THS1		
RPS42		GCN1		RNR21		
C5_00030W		ARP7		GLN3		
CDC19		HOG1		C7_03840W		
RNR1		HET1		CLC1		
RPS26A		C5_00260W		C6_02880W		
ENO1		NSA1		ZCF29		
CR_00460C		CSI2		PMM1		
YDJ1		PWP1		UTP5		
ADE4		C6_02350C		C2_07220W		
RPS13		SEC26		C3_04810C		
SMT3		GLC7		CR_02420W		
C4_05630W		PIM1		CCJ1		
PDB1		VMA5		MRPL10		
ACS2		CR_03200C		MYO2		
C1_12280C		SRP40		DPS1-1		
KAR2		C1_03790C		RAS1		
C1_00160C		DNM1		C3_06700C		
PGK1		C1_02240W		C5_00080C		
C2_04570W		PRO2		C2_07290W		

bioRxiv preprint doi: https://doi.org/10.1101/2025.06.23.660276; this version posted June 26, 2025. The copyright holder for this preprint (which was not certified by peer review) is the author/funder, who has granted bioRxiv a license to display the preprint in perpetuity. It is made available under aCC-BY 4.0 International license.

PRT1		PMA1		HXK2		
TKL1		RPD3		C3_07420W		
LAT1		C6_02370C		MSS116		
NIP1		C1_09040C		MRPS9		
ARO8		PNG2		CSH1		
C4_04820C		C1_00060W		GRP2		
TAF14		RPA190		ARC35		
LYS22		MSI3		NMD3		
HSP21		UTP8		C5_00790C		
MRPL3		TOM1		CHA1		
PIL1		ERB1		KRR1		
PDX1		FAS2		LYS21		
HIS7		PFK1		AGO1		
FAS1		MET10		C6_04100W		
SER33		MDN1		C5_04640C		
URA2		C2_02540W		ERG6		
SDH12				RNR22		
SUB2				LMO1		
PES1				SWD1		
PET9				SES1		
RPG1A				C1_12240C		
SEC27				MIS11		
PFK2				C6_03440W		
CDC48				ARO9		
C3_02350W				AIP2		
ATP2				HOM3		
ILV2				PBS2		
CHC1				GAD1		
PDA1				HAS1		
C1_00930C				GGA2		
GLT1				APE2		
TIF4631				C1_00640C		
HSP104				C2_06200C		
SPT6				ROB1		
SNF12				RGD1		
LPD1				VPS1		
NAN1				C1_08110W		
PGA63				MSN4		
ACC1				C7_03160W		
DED1				RRP6		
PPH21				NUP82		
C1_05630C				SEC12		
C2_04370W				C1_14080W		
SHM2				CRM1		
C5_02380W				PEX1		
KRE30				CCT6		
C1_01590C				PYC2		
CDC53				C7_01030C		
C4_00420C				SNF2		
				NAB3		

bioRxiv preprint doi: https://doi.org/10.1101/2025.06.23.660276; this version posted June 26, 2025. The copyright holder for this preprint (which was not certified by peer review) is the author/funder, who has granted bioRxiv a license to display the preprint in perpetuity. It is made available under aCC-BY 4.0 International license.

				SPO72		
				CDC39		
				SSN6		
